# Bizarre intravascular leiomyoma with intracardiac extension starting in the ovarian vein: a case report from Syria

**DOI:** 10.1093/omcr/omae131

**Published:** 2024-11-20

**Authors:** Amal Babi, Baraa Shebli, Mike Ghabally, Hussein Alkanj

**Affiliations:** Department of Echocardiography, Aleppo University Hospital, University of Aleppo, Aleppo, Syria; Cardiology Division, Internal Medicine Department, Aleppo University Hospital, University of Aleppo, Aleppo, Syria; Cardiology Division, Internal Medicine Department, Aleppo University Hospital, University of Aleppo, Aleppo, Syria; Cardiac Surgery Department, Aleppo University Hospital, University of Aleppo, Aleppo, Syria

**Keywords:** cardiac, tumor, mass, intravascular leiomyoma, intracardiac extension, pulmonary, syncope

## Abstract

Intravascular leiomyoma (IVL) with intracardiac extension (ICE) represents an exceedingly rare diagnosis of a cardiac mass. We present the case of a 42-year-old woman with recurrent syncopal episodes. Cardiac investigations revealed an extensive, mobile mass stretching from the inferior vena cava (IVC) through the right heart to the bifurcation of the pulmonary artery. Emergent surgery was conducted to excise the mass. Post-operative assessment indicated a potential malignancy in the adnexa. A subsequent surgery to resect the uterus with the adnexa, the primary origin of the mass, confirmed the diagnosis of IVL with ICE. The initial diagnostic ambiguity and the urgent pulmonary artery involvement necessitated a two-step surgical approach. Despite the propensity for recurrence, a 5-year follow-up remained unremarkable. This case underscores the importance of considering IVL with ICE in the differential diagnosis, which can expedite both diagnosis and treatment.

## Introduction

Intravascular leiomyoma (IVL) is an exceptionally rare, histologically benign neoplasm originating from uterine smooth muscle cells, yet it displays clinical aggressiveness. Typically, tumor cells infiltrate the uterine vascular spaces, advancing into the venous system, and may subsequently access the iliac vein and inferior vena cava (IVC) through the uterine and ovarian veins [1]. Occasionally, the tumor extends into the right heart chambers, with pulmonary artery involvement observed in rare instances.

Extra-uterine extension involving the right heart chambers is termed Intravascular Leiomyoma with Intra-cardiac Extension (ICE) or Intra-cardiac Leiomyomatosis (ICL) [1, 2]. While fewer than 300 cases of IVL are documented in the medical literature, there is an increasing trend in reported ICE cases, approaching almost 100 [3]. However, pulmonary artery involvement is estimated at 8.8% in cases with ICE [4].

Diagnosing and managing IVL with cardiac extension poses significant challenges, demanding specialized expertise. Clinical presentations are often nonspecific, and radiological features can be inconclusive [3, 5]. IVL exhibits diverse pathological subtypes, including cellular, myxoid, and bizarre forms [4]. Herein, we present a case of the rare bizarre subtype of IVL with ICE, initially manifested as syncope, and discuss its unique aspects in light of the existing literature.

## Case report

A 42-year-old woman presented to the emergency department with syncope and urinary incontinence. She had experienced multiple syncopal episodes over the past three years without prior medical evaluation. Notably, she underwent surgical excision of a uterine leiomyoma two years ago and recently had a laparoscopy as part of infertility investigations.

**Figure 1:**
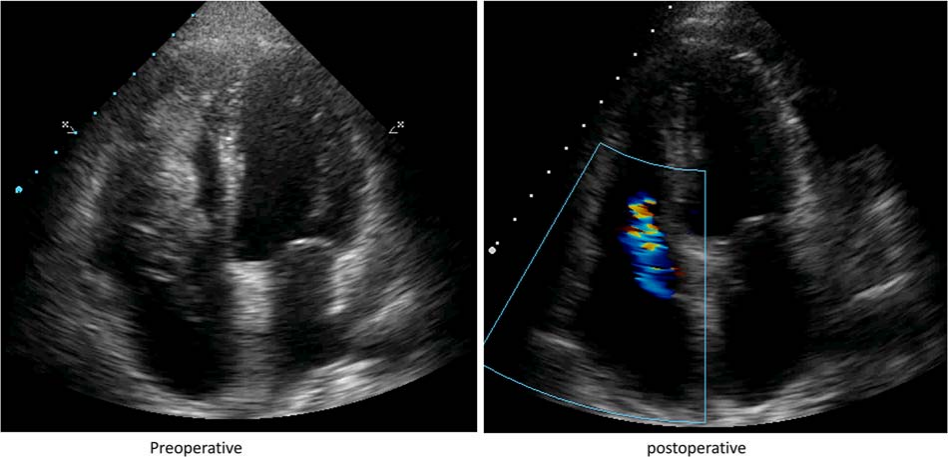
Transthoracic echocardiography revealed the presence of an extremely mobile, lobulated cardiac mass protruding from the right atrium through the tricuspid valve into the right ventricle (image left). The image on the right side shows a postoperative view, highlighting the changes found after the surgical intervention.

Upon assessment, her vital signs were within normal ranges. Cardiac examination revealed a 2/6 murmur over the tricuspid area, and examination of the extremities identified the presence of purpura. Electrocardiography (ECG) displayed biphasic T waves in leads V3-V4. Cardiac echocardiography (Fig. 1) revealed:

- An echogenic, floating, stalkless, freely-mobile mass extending from the inferior vena cava (IVC) to the right atrium and passing through the tricuspid valve into the right ventricle.
- Mild tricuspid regurgitation, along with pulmonary arterial hypertension measuring 42 mmHg systolic.

**Figure 2:**
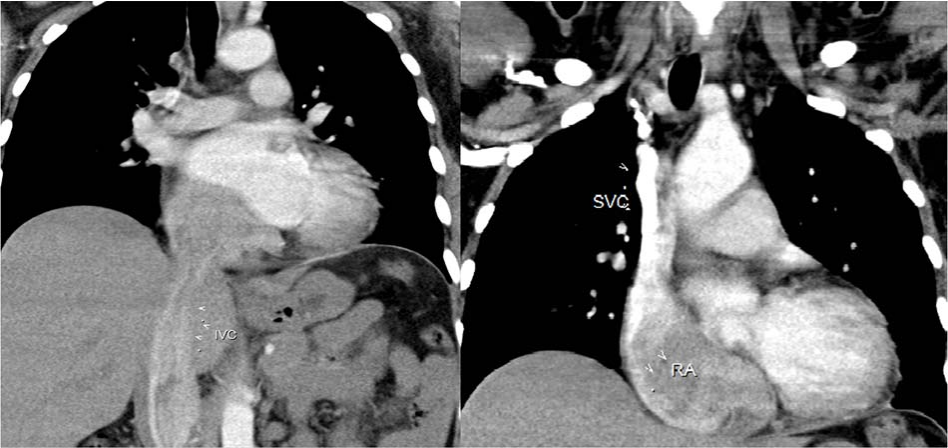
Two different MSCT images that shows the extent of the tumour.

Abdominal echocardiography and Doppler study of the lower extremities’ veins identified a floating mass within the inferior vena cava (IVC), revealing the mass’s extension through the IVC, originating just above the renal veins. The mass filled the right atrium and partially traversed the tricuspid valve. Remarkably, MSCT disclosed a non-obstructing tumor extension into the right pulmonary artery, just before the bifurcation at the hilar region (Fig. 2).

The patient was admitted immediately and assessed for emergent surgical intervention. Basic laboratory tests and D-dimer levels were normal. Full coagulation profile was within normal ranges. ANA, Anti ds-DNA, TSH, CEA, and CA-125 were also normal except for a high CA-125 level (145.2 μ/l; normal range: up to 35 μ/l), indicating a potential malignant origin of the lesion.

**Figure 3:**
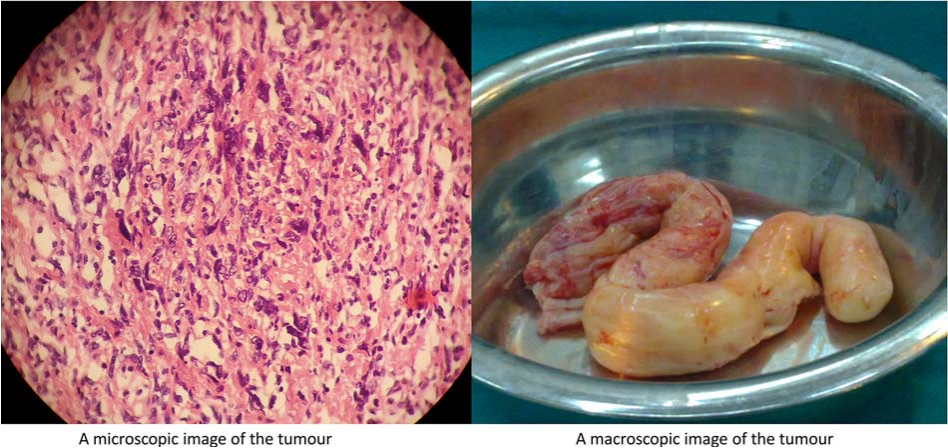
Microscopic image showing spindle cells with scattered bizarre nuclei and absence of abnormal mitotic figure, extending within the muscular layer (left). The image on the right shows the appearance of the tumour after the resection.

Subsequently, the patient underwent emergency surgery to excise and assess the lesion. A median sternotomy was performed, opening the right atrium and revealing a large mass extending through the IVC. Due to the mass’s immobility, a laparotomy was conducted. Intraoperative examination identified the mass originating from the ovarian vein and extending throughout the IVC. Proper incisions were made to remove the mass. Pathological examination of the excised specimen was inconclusive, necessitating immunohistochemical techniques for further evaluation (Fig. 3). Post-operative cardiac echocardiography showed normal results, except for residual mild tricuspid regurgitation (TR) with normal systolic pulmonary pressure.

Comprehensive postoperative investigations, including abdominal ultrasonography, pelvic MRI, and abdominal MSCT, revealed a multi-lobulated mass in the right adnexa extending to the body and fundus of the uterus, exhibiting intense contrast agent enhancement.

Given the strong indications of ovarian malignancy, the patient subsequently underwent a subtotal hysterectomy with bilateral salpingo-oophorectomy. Pathological studies confirmed the diagnosis of bizarre intravascular leiomyomatosis with cardiac extension. Despite the elevated recurrence rates associated with this condition, a 5-year postoperative clinical and radiological follow-up remained uneventful.

## Discussion

Intravascular Leiomyomata (IVL) is an extremely rare benign tumor that was first described by Birch-Hirschfeld in 1986 [6]. Less than 300 cases of IVL were reported in the medical literature, with less than 100 cases of Intra-cardiac extension (ICE). Nevertheless, pulmonary involvement was only reported in 8.8% of cases of IVL with ICE [4].

We reported a case of a patient with recurring obstructing symptoms, diagnosed as IVL with ICE. The pulmonary involvement -as reported in this manuscript- is not only very seldom reported in the literature, it is considered an emergency situation.

Intravascular Leiomyomata (IVL) is an exceedingly rare benign tumor initially described by Birch-Hirschfeld in 1986 [6]. The medical literature reports fewer than 300 cases of IVL, with Intra-cardiac extension (ICE) documented in less than 100 cases. Nevertheless, pulmonary involvement is reported in only 8.8% of IVL cases with ICE [4].

This manuscript presents a case of a patient experiencing recurrent obstructive symptoms, diagnosed with IVL with ICE. Aside from being infrequently documented, the pulmonary involvement highlighted in this report is considered an emergency situation.

The average age at diagnosis is 45 years, with the key risk factor being a history of leiomyomata, myomectomy, or hysterectomy [3, 7]. IVL typically infiltrates the pelvic venous system, progressing through the uterine and ovarian veins to the internal iliac, common iliac, and renal veins, ultimately reaching the IVC [4].

Clinical manifestations vary based on factors such as tumor size, extension beyond the uterus, and the presence of complications. These might be attributed to the primary pelvic tumor, IVC infiltration, tricuspid valve or pulmonary valve occlusion (as in this case), or complications [3, 7].

The pre-operative diagnosis of IVL with ICE presents challenges. Transthoracic echocardiography (TTE), typically the initial method, demands specialized expertise [1, 7]. Trans-esophageal echocardiography (TEE) proves valuable for a detailed assessment of the tumor’s relationship with heart valves and chambers [1, 5, 7]. IVL with ICE typically manifests as an echogenic, floating, stalkless, freely-mobile mass [1, 2, 7]. The most common valvular disturbance observed is mild to moderate tricuspid regurgitation, often remaining mild post-tumor resection, as seen in our case [7].

TEE surpasses TTE in evaluating right atrial tumors, and intraoperative TEE is recommended, if available, before tumor retraction to minimize vascular injury and ensure complete removal [7, 8]. However, in our case, intraoperative TEE could not be performed due to limited available resources.

Differential diagnoses are diverse and include, but are not limited to, intravascular thrombosis, Budd-Chiari syndrome, right atrial myxoma, and endometrial stromal sarcoma, posing a diagnostic challenge in some cases [3, 5, 7, 8]. This complexity might have initially jeopardized a prompt and straightforward diagnostic process in our case.

The primary treatment approach involves complete tumor resection along with total hysterectomy and bilateral salpingo-oophorectomy. The surgery can be carried out as a one- or two-step procedure. While a single-stage approach is preferable, a two-stage surgery is reserved for patients unable to tolerate extensive surgery [3, 7, 8]. In this case, a two-step procedure was performed, due to the initially inconclusive diagnosis. Histological and immunohistochemical studies are the cornerstone of the definitive diagnosis [2, 7, 8]. IVL has many pathological subtypes, including cellular, myxoid, and bizarre; with the bizarre subtype that is reported in our case being one of the rarely reported subtypes [3].

As this clinical entity is very rare, the diagnosis might be very challenging if the clinician has not kept it in mind, otherwise the diagnosis might be missed initially. In our case; given the emergent type of the procedure and investigation, thorough evaluation was not possible and the diagnosis was overlooked at first. We advise clinicians and cardiologists to keep IVL with ICE in mind as an important differential diagnosis when dealing with similar findings on echocardiography, as this might aid and accelerate the diagnostic process and affect the management dramatically.

Due to the rarity of this clinical entity, the diagnosis can be exceptionally challenging if not actively considered by the clinician, potentially leading to initial oversight. In our case, the urgency of the procedure and investigation precluded a comprehensive evaluation, resulting in the initial omission of the diagnosis. We strongly recommend that clinicians and cardiologists be vigilant and consider IVL with ICE as a crucial differential diagnosis when confronted with similar echocardiographic findings. This proactive approach can significantly expedite the diagnostic process and have a substantial impact on management decisions.
